# Assessment of Oral Conditions and Quality of Life in Morbid Obese and Normal Weight Individuals: A Cross-Sectional Study

**DOI:** 10.1371/journal.pone.0129687

**Published:** 2015-07-15

**Authors:** Joselene Martinelli Yamashita, Patrícia Garcia de Moura-Grec, Adriana Rodrigues de Freitas, Arsênio Sales-Peres, Francisco Carlos Groppo, Reginaldo Ceneviva, Sílvia Helena de Carvalho Sales-Peres

**Affiliations:** 1 Department of Pediatric Dentistry, Orthodontics and Public Health, Bauru School of Dentistry, University of São Paulo, Bauru, SP, Brazil; 2 Department of Surgery and Anatomy, School of Medicine of Ribeirão Preto, University of São Paulo, Ribeirão Preto, SP, Brazil; 3 Department of Physiological Sciences, Piracicaba Dental School, University of Campinas, Piracicaba, São Paulo, Brazil; University of North Carolina at Chapel Hill, UNITED STATES

## Abstract

The aim of this study was to identify the impact of oral disease on the quality of life of morbid obese and normal weight individuals. Cohort was composed of 100 morbid-obese and 50 normal-weight subjects. Dental caries, community periodontal index, gingival bleeding on probing (BOP), calculus, probing pocket depth, clinical attachment level, dental wear, stimulated salivary flow, and salivary pH were used to evaluate oral diseases. Socioeconomic and the oral impacts on daily performances (OIDP) questionnaires showed the quality of life in both groups. Unpaired Student, Fisher’s Exact, Chi-Square, Mann-Whitney, and Multiple Regression tests were used (p<0.05). Obese showed lower socio-economic level than control group, but no differences were found considering OIDP. No significant differences were observed between groups considering the number of absent teeth, bruxism, difficult mastication, calculus, initial caries lesion, and caries. However, saliva flow was low, and the salivary pH was changed in the obese group. Enamel wear was lower and dentine wear was higher in obese. More BOP, insertion loss, and periodontal pocket, especially the deeper ones, were found in obese subjects. The regression model showed gender, smoking, salivary pH, socio-economic level, periodontal pocket, and periodontal insertion loss significantly associated to obesity. However, both OIDP and BOP did not show significant contribution to the model. The quality of life of morbid obese was more negatively influenced by oral disease and socio-economic factors than in normal weight subjects.

## Introduction

Obesity is defined as a chronic disease affecting the general health due to the abnormal or excessive body fat accumulated [1, 2]. Nutritional habits, technological advancements, and industrialized food associated with sedentary lifestyle have been related as the main causes of the epidemic obesity [3, 4]. The origin of obesity is recognizably multifactorial and related with the interaction of genetic, metabolic, social, cultural and behavioral factors [5].

Obesity is a worldwide epidemic affecting not only developed countries, but also the developing world, where a significant increase in overweight and obesity has been observed [6]. Many of obesity consequences increase the risk of premature death [7], and directly affect the quality of life of affected subjects [5, 8]. Furthermore, it increases comorbidities and complications in both systemic and oral health [9].

Coronary heart disease, hypertension and stroke, certain types of cancer, diabetes, gallbladder disease, dyslipidemia, osteoarthritis, gout, and pulmonary diseases, including sleep apnea [10] are some comorbidities directly related to obesity. Obesity has been associated with oral conditions such as periodontal disease [11, 12], dental caries [13], dental wear [14, 15] and xerostomia [16]. As part of overall general health [9], oral health is relevant in primary prevention and risk-reduction strategies [9, 17, 18].

Considering the ever-increasing prevalence of obesity and the possible relationship between obesity and oral disease, the aim of this study was to evaluate the impact of oral disease on the quality of life in both morbid obese and normal weight subjects.

## Materials and Methods

### Subjects

Sample size was calculated by using Statistica software (StatSoft Inc., Tulsa, USA), considering significance level set at 5%, 80% of power, and a sample ratio of 2:1, which resulted in 96 subjects in the experimental group. A cross-sectional study was designed with 100 (23 males and 77 females) morbid obese subjects (obese group) during preoperative care for surgery, and 50 (6 males and 44 females) normal-weight subjects (control group). Subjects were recruited and examined at the Clinical Hospital at the School of Medicine of Ribeirão Preto–USP from 2012 to 2013.

The study was performed according to the Declaration of Helsinki guidelines, being all procedures approved by the Ethics and Research Committee of the Bauru School of Dentistry, University of São Paulo, Brazil (Proc. n°196/2011). All subjects signed a written consent form regarding their participation in the study.

### Anthropometric assessment

Individual body mass index (BMI) was obtained by dividing the subjects’ weight (in kg) by their height raised to the power of 2 (in m^2^). Weight was obtained in an automatic weighing apparatus (MIC model 300PP, Micheletti Ind., max capacity 300 kg), and height in a stadiometer (Wood 2.20, WCS Ind., Brazil). Subjects were considered obese when BMI was ≥ 40.0 kg/m² and normal-weight when BMI 18.5 to 24.9 kg/m² [10].

### Socioeconomic and quality of life surveys

All subjects were submitted to a socioeconomic survey and to the Oral Impacts on Daily Performances (OIDP), which is used to determine quality of life conditions.

The socioeconomic questionnaire included 5 questions regarding 1) monthly family income; 2) number of family members; 3) education level; 4) housing status; and 5) occupation. To each question, a number from 0 to 10 was assigned according to previous defined scores [19–21]. After summing the scores of those five questions, the total score was used to classify the subjects into five socioeconomic ranks: 1) high (final score from 9 to 10); 2) average-high (final score from 7 to 8.9); 3) average (final score from 5 to 6.9); 4) average-low (final score from 3 to 4.9); and 5) low (final score from 1 to 2.9).

The OIDP survey was used to observe the how the oral conditions influenced the subjects to perform the following activities on the past 6 months: 1) physical activities—eating and enjoying food, speaking and pronouncing clearly, cleaning teeth; 2) psychological activities—sleeping and relaxing, smiling and showing teeth without embarrassment, maintaining usual emotional state; and 3) social activities—carrying out major work and social activities, enjoying contact with other people [22]. A score considering the frequency (1 to 5) and the severity of the impact (0 to 5) was attributed to each question. The frequency was then multiplied by the severity in order to stablish a score for each one of the three activities. The total OIDP score was the sum of scores of all activities (physical + psychological + social), and sums different from zero were classified according to the following distribution: 1) low (1–15); 2) medium (16–45); and 3) high (46–200). In addition, OIDP of each subject was classified as “0” for the subjects without impact (OIDP = 0); and as “1” for those subjects with impact (OIDP > 0).

Besides the anthropometric and socioeconomic/quality-of-life assessments, information regarding smoking, hypertension, and diabetes was also obtained. Only well-controlled subjects with hypertension and/or diabetes were admitted in the study. These conditions were controlled by using different drug combinations. Since different drug protocols were used, this information was not addressed in the statistics.

### Dental examination

All oral examinations were conducted by a previously calibrated dentist (Kappa > 0.90). This clinical examination evaluated dental caries according to the International Caries Detection & Assessment System II—ICDAS II [23].

Community Periodontal Index (CPI) was used to evaluate gingival bleeding on probing (BOP), calculus, and probing pocket depth (PPD). The Clinical Attachment Level (CAL) was used to assess periodontal diseases, estimating the cumulative destruction of the teeth supporting tissues, in accordance to the World Health Organization (WHO) [24].

Both prevalence and severity of dental wear were evaluated according to the Dental Wear Index (DWI) [25]. Salivary flow was measured according to the circadian cycle (from 9 to 11 am) by collecting stimulated saliva, which was obtained directly from subjects every minute, after they chew a rubber-band piece for 5 min. The flow was considered as “low” when under 1mL/min [26]. Salivary pH was measured by pH strips (MerckMerck/EMD, São Paulo, Brazil), being pH 6 considered acid, 7 normal, and pH 8 alkaline [27].

### Data analysis

All statistical analyses were carried out by Systat 13.0, BioEstat 5.0, and GraphPad 6.0. Shapiro-Wilk´s and Levene´s tests were used to observe data normality and homoscedasticity of variances, respectively. Differences between groups were analyzed by Unpaired Student´s *t* (with or without Welch´s correction), Chi-square (with Yates´s correction), Fisher´s Exact, and Mann-Whitney´s tests. In addition, Spearman´s test was used to observe possible correlation among some variables. Direct dependency between obesity and categorical data was assessed by a logistic regression. A significance level of 5% was set for all tests.

## Results

Table 1 shows the demographics and the anthropometric assessment of both groups. Obese volunteers where older and heavier than the ones in control group, but no significant differences were observed between their heights. Thus, the main component of BMI of both groups was the volunteers´ weight.

**Table 1:** Demographics and the anthropometric assessment of both groups.

	*Control (n = 50)*	*Obese (n = 100)*	*p*	*Test*
**Age in years (mean±SEM)**	31.3 (± 1.3)	37.6 (±1.0)	0.0007	Unpaired t
**Weight in kg (mean±SEM)**	59.6 (±1.3)	138.1 (±2.9)	< 0.0001	Unpaired t/Welch correction
**Height in meters (mean±SEM)**	1.65 (±0.01)	1.65 (±0.01)	0.83	Unpaired t
**BMI in kg/m^2^ (mean±SEM)**	21.9 (±0.3)	50.9 (±0.9)	< 0.0001	Unpaired t/Welch correction
**Female**	44 (88%)	77 (77%)	0.17	Chi-square/Yates correction
**Male**	6 (12%)	23 (23%)		
**Smoker**	9 (18%)	7 (7%)	0.08	Chi-square/Yates correction
**Non-Smoker**	41 (82%)	93 (93%)		
**Hypertension**	2 (4%)	58 (58%)	< 0.0001	Fisher´s Exact
**Non-Hypertension**	48 (96%)	42 (42%)		
**Diabetes**	-	24 (24%)	< 0.0001	Fisher´s Exact
**Non-Diabetes**	50 (100%)	76 (76%)		
**Socio-economic level index (mean±SEM)**	6.6 (±0.26)	5.1 (±0.15)	< 0.0001	Unpaired t
**Low socio-economic level**	2 (4%)	6 (6%)	< 0.0001	Chi-square/Yates correction
**Low to average socio-economic level**	6 (12%)	40 (40%)		
**Average socio-economic level**	19 (38%)	44 (44%)		
**Average to high socio-economic level**	19 (38%)	9 (9%)		
**High socio-economic level**	4 (8%)	1 (1%)		
**Total OIDP (median/interquartile deviation)**	0 (3)	0 (15)	0.10	Mann-Whitney
**No impact**	35 (70%)	58 (58%)	0.09	Chi-square/Yates correction
**Low OIDP**	10 (20%)	19 (19%)		
**Average OIDP**	4 (8%)	10 (10%)		
**High OIDP**	1 (2%)	13 (13%)		

Gender proportions did not differ between groups, but there were more females in both groups. In fact, the proportions between females and males followed a 4:1 ratio in both control (p = 0.17) and obese (p = 0.57) groups. No differences regarding smoking habits were found between groups, and smokers consumed similar quantity of cigarettes/day in both groups (control = 9.1±1.6; obese = 8.7±0.6, p = 0.53).

As expected, both hypertension (Odds ratio = 33.1, p<0.0001) and diabetes (Odds ratio = 32.5, p<0.0001) were more prevalent in obese than in control volunteers.

A lower measurement of the socio-economic level was observed in obese than in control group. In addition, no significant differences were observed between groups in both proportions of high and low socio-economic levels, but higher proportions of average-to-low socio-economic levels were observed in obese volunteers. No significant differences were observed between groups considering OIDP.

Table 2 shows no significant differences between groups considering the number of absent teeth, bruxism, difficult mastication, calculus, initial caries lesion, and caries. However, salivary flow was low in obese volunteers. The salivary pH was also different in obese, presenting a tendency to be lower (p = 0.0028) or higher (p = 0.0017) than the control volunteers. Enamel wear was lower in obese, but dentine was higher than controls. Obese group exhibited more BOP and periodontal pocket, especially the deeper ones, than the control group. Insertion loss (CAL) was also more frequent in obese volunteers.

**Table 2:** Oral health conditions of both groups.

	*Control (n = 50)*	*Obese (n = 100)*	*p*	*test*
**Salivary flow (median/interquartile deviation)**	0.9 (0.55)	0.6 (0.59)	0.0024	Mann-Whitney
**Number of absent teeth (median/interquartile deviation)**	4 (3)	4 (6)	0.10	Mann-Whitney
**Number of teeth with enamel alterations (median/interquartile deviation)**	3.5 (6)	3 (6)	0.97	Mann-Whitney
**Enamel wear (median/interquartile deviation)**	26.5 (8.8)	21.0 (9.5)	0.0027	Mann-Whitney
**Dentine wear (median/interquartile deviation)**	5.2 (8.1)	12.5 (13.1)	0.0004	Mann-Whitney
**Total wear (median/interquartile deviation)**	33.3 (0.7)	33.3 (1.4)	0.31	Mann-Whitney
**Salivary pH 6**	6 (12%)	30 (30%)	<0.01	Chi-square/Yates correction/ Fisher´s Exact
**Salivary pH 7**	40 (80%)	44 (44%)		
**Salivary pH 8**	4 (8%)	25 (25%)		
**No bruxism**	43 (86%)	79 (79%)	0.46	Chi-square/Yates correction
**Bruxism**	7 (14%)	21 (21%)		
**No difficult mastication**	43 (86%)	77 (77%)	0.28	Chi-square/Yates correction
**Difficult mastication**	7 (14%)	23 (23%)		
**No bleeding on probing**	38 (76%)	45 (45%)	0.0006	Chi-square/Yates correction
**Bleeding on probing**	12 (24%)	55 (55%)		
**No calculus**	5 (10%)	3 (3%)	0.12	Fisher´s Exact
**Calculus**	45 (90%)	97 (97%)		
**No periodontal pocket**	40 (80%)	43 (43%)	< 0.0001	Chi-square/Yates correction
**Periodontal pocket**	10 (20%)	57 (57%)		
**CPI—Sound teeth**	5 (10%)	2 (2%)	< 0.0001	Chi-square/Yates correction
**CPI—Calculus**	36 (72%)	41 (41%)		
**CPI—PPD 4 to 5 mm**	9 (18%)	49 (49%)		
**CPI—PPD 6 mm or more**	0 (0%)	8 (8%)		
**CAL between 0 and 3 mm**	49 (98%)	69 (69%)	0.0001	Chi-square/Yates correction
**CAL between 4 and 5 mm**	1 (2%)	18 (18%)		
**CAL between 6 and 8 mm**	(0%)	12 (12%)		
**CAL between 12 mm or more**	(0%)	1 (1%)		
**No initial caries lesion**	9 (18%)	10 (10%)	0.26	Chi-square/Yates correction
**Initial caries lesion**	41 (82%)	90 (90%)		
**No caries**	41 (82%)	66 (66%)	0.07	Chi-square/Yates correction
**Caries**	9 (18%)	34 (34%)		

Possible correlations among BMI and the other factors are shown in Table 3. All correlations, except between BMI and total wear, were significant (p<0.05), but all of them were weak (rS < 0.4). Interestingly, both salivary flow and economical level were inversely proportional to BMI.

**Table 3:** Correlation among BMI and the other factors observed.

	*Spearman Ro (rS)*	*p*
**Salivary flow**	-0.21	0.0111
**Economical level**	-0.41	<0.0001
**OIDP**	0.24	0.0031
**Enamel wear**	-0.28	0.0005
**Dentine wear**	0.33	<0.0001
**Total wear**	0.05	0.5155

The logistic regression is shown in Table 4. The following dichotomized variables were included to construct a model with adjustment for age: gender, smoking, salivary flow (1 = hyposalivation; 0 = normal flow), salivary pH (1 = pH 6 or 8; 0 = pH 7), socio-economical level (0 = low; others = 1); OIDP (without impact = 0; with impact = 1), BOP (1 = with BOP), calculus (1 = with calculus), periodontal pocket (1 = with at least one pocket), CPI (0 = 0, others = 1); and CAL (1 = with periodontal insertion loss). The best model was obtained after excluding salivary flow, calculus, and CPI. The model was significantly better than by chance to predict obesity (log-2 likelihood = 115.8, p < 0.0001). The Nagelkerke´s R^2^ showed that the variables (except salivary flow, calculus, and CPI) are responsible for approximately 55% of variation of obesity. Hosmer & Lemeshow´s test showed the validity of the model (Chi-square = 4.629; p = 0.796), and the overall percentage of prediction obtained was 80%, considering a cutoff of 0.5.

**Table 4:** Logistic regression parameters among obesity and other categorical data.

						IC95% of EXP(B)	
	B	S.E.	Wald	Sig. (p)	Exp(B)	Lower	Upper
Gender	-1.42	0.64	4.98	0.0256	0.24	0.07	0.84
Smoking	2.63	0.86	9.4	0.0021	13.93	2.59	75.05
Salivary pH	-2.07	0.56	13.61	0.0002	0.13	0.04	0.38
Socio-economical level	1.84	0.58	9.95	0.0016	6.3	2.01	19.76
OIDP	-0.74	0.54	1.85	0.1743	0.48	0.16	1.39
BOP	-0.48	0.53	0.81	0.3689	0.62	0.22	1.76
Periodontal pocket	-1.75	0.57	9.43	0.0021	0.17	0.06	0.53
CAL	-2.77	1.14	5.87	0.0153	0.06	0.01	0.59
Constant	4.51	1.57	8.28	0.0040	90.67		

The model shows that all predictors, except for OIDP and BOP, significantly contribute to obesity. The most important predictors in the model were smoking and socio-economical level. Low socio-economical level and smoking habit increased very much the odds for obesity.

## Discussion

The main limitation of the present study was the difficult matching between subjects in both groups, regarding age and the socioeconomic status. However, logistic regression was designed to minimize this limitation.

Approximately 17% of Brazilian women are obese, while approximately 12% of men exhibits this condition [28]. The proportion between females and males in the present study did not reflect the Brazilian proportion between obese women and men, since the sample had more females (77%) than males. Usually, women seek more often the surgical treatment, probably because they care more about health and they are more unsatisfied with their body shape. Besides, it is well-known that the excess of weight could decrease the quality of life, being the image of thinness constantly promoted by media as a symbol of sexuality, beauty and success [29, 30]. Men prefer to use other services, such as pharmacies and emergency rooms than doctors in clinics or hospitals [31].

The “average” level prevailed as the socioeconomic status for both groups. However, the obese subjects showed a tendency to the lower socioeconomic levels, while the control subjects showed a tendency to the higher ones. The sample consisted mainly of subjects seeking the Brazilian public health system, which provides treatment (including bariatric surgery) for obesity. In Brazil, individuals with high-socioeconomic status have better health plans, more access to healthy food and physical activity. The opposite seems to be true for those individuals with low-socioeconomic status, which have more consumption of animal foods, saturated fat, sugar, and less time for physical activity [32]. Thus, the socioeconomic status observed in the obese group was expected to be lower than their matching counterparts in the control group.

As expected, the obese group had a high percentage of individuals with hypertension. In fact, obesity is as an important risk factor for hypertension, causing increased blood pressure regardless the sodium intake [12, 33]. Effective treatments resulting in weight control have also reduced the blood pressure [34, 35]. Besides hypertension, obesity is a risk factor for diabetes [36]. The association between diabetes and periodontal disease progression was also previously studied. Hyperglycemia seems to cause structural alterations in periodontal tissues and impairment of the immune cells response [37, 38]. All the diabetic and hypertensive subjects in the present study were receiving drugs to treat their condition. No effect of hypertension was observed in any of the periodontal disease parameters in the obese group.

Psychological malaise, low self-esteem and associated comorbidities were previously pointed out as factors affecting the quality of life of obese patients [39]. However, in the present study, the quality of life measured by psychological aspects of OIDP survey did not differ between groups. In fact, none of the IODP survey components (data not shown) differed between obese and normal-weight subjects. Bariatric surgery has been associated with a decreased psychopathology rather than decreased body-weight and increased physical function [40]. In our study, obese subjects were close to the bariatric surgery intervention, and being treated by a multi-professional medical team, which included phycologists and psychiatrists. This medical care along with the subjects expectation could have contributed to the decreased psychological disorders and improved quality of life observed in the obese subjects of our study.

The association between obesity and dental caries remains controversial. A previous study showed obesity as a possible predictor for dental caries due to a high sugar ingestion in obese subjects [13]. In the present study, no differences between groups were observed regarding either initial or deeper caries lesions. Studies correlating obesity and dental caries in adults are scarce, and usually contradictory. For instance, Isaksson (2013) [41] and Östberg et al. (2012) [42], both studying Swedish adult obese and non-obese subjects, showed different conclusions. The first study observed obese individuals with more caries than the normal individuals, while the second study observed no association between the number of carious lesions and obesity.

Similarly, the association between obesity and periodontal disease is still unclear [12, 43–48]. Our results showed more severe periodontal disease (PPD, BOP, and CAL) in the obese group. Obesity causes “hyper-inflammatory” state, characterized by increased number of macrophages, leukocytes, and lymphocytes. These cells infiltrate in the adipose tissue, activating cytokines, which impair the local immune response [49]. Additionally, diabetes, which was present only in obese subjects in our study, has previously shown a great relationship with periodontal disease [37, 38], and it could be a confounder effect during data analysis. However, comparison between non-diabetic and diabetic obese subjects revealed no differences (data not shown) regarding any periodontal parameter (BOP, PPD, calculus, CAL, and CPI). Difficulty teeth’s cleaning was also not significant in the obese group, and it probably did not influence the periodontal disease verified in those subjects. Thus, it is possible that inflammatory state caused by obesity itself could be responsible for the periodontal disease severity observed here.

Dental wear (enamel + dentine wear) did not differ between groups, but dentine wear was more severe in the obese group, while enamel wear was more evident in the control group. Association between dental erosion and overweight/obesity in adults was previously reported [41]. Many factors, such as gastroesophageal reflux, bruxism, salivary pH, and age, may have influenced this finding. Other studies showed a direct relationship between aging and dental wear [50, 51]. In fact, in our study, direct correlations were observed between dentine wear with both age (data now shown) and BMI. Interestingly, the correlations between enamel wear and age (data now shown) and BMI were both inverse.

Obesity is considered a risk factor for gastroesophageal reflux [52], which may cause dental erosion [53]. Despite the absence of data on gastroesophageal reflux episodes on the obese group, both consumption of acid food and reflux could be responsible for most of dental erosion in obese subjects. Anxiety is also associated with obesity [54], and it could cause episodes of bruxism [55], and dental attrition [53, 56]. In our study, subjects with bruxism showed more dentine wear, but no influence of bruxism was observed in enamel wear.

Salivary pH could also affect the dental erosion rate [57], and low salivary pH tended to be more related (data not shown) to both dentine and enamel wear in the present study. In addition, salivary flow rate was lower in obese subjects, which could contribute to periodontal disease and dental wear [14, 58–61]. Among many other causes, changes in salivary flow rate are attributed to drugs used to treat many systemic diseases, such as diabetes, hypertension, and depression [62]. In our study, the subjects in treatment to control hypertension showed low salivary flow rates, but diabetics did not show significant changes in salivary flow when compared to the non-diabetic subjects.

Despite the limitation on salivary pH analysis, which was measured by pH-strips with a limited degree of accuracy, obese tended to show variation in pH of saliva. In the obese subjects, slightly acid or slightly alkaline pHs were more frequent. In fact, salivary pH is affected by obesity, especially when metabolic syndrome is present, being the degeneration of acinar cells frequently related in diabetic and dyslipidemic subjects [63].

Our logistic regression model showed some variables with a significant impact in obesity. Among those variables, some of them such as gender, smoking, OIDP, and socio-economical level were not susceptible or less susceptible to changes. However, periodontal diseases (BOP, periodontal pockets, and CAL) and oral conditions (salivary pH) are subject to clinical intervention. The relationship between obesity and periodontal disease, irrefutably showed by logistic regression, was previously suggested by others authors [56, 64, 65].

Metabolic disturbances caused by obesity impair the general health, affecting virtually all organic systems [8, 66]. As showed in our study, oral diseases may also be part of the metabolic condition in obese subjects, compromising the general health of these individuals. Thus, oral care programs design for obese patients, implemented with other multidisciplinary care, could have a direct and positive impact in their general health.

## Conclusion

The quality of life of morbid obese was more negatively influenced by oral disease and socio-economic factors than in normal weight subjects.

## Supporting Information

S1 File Distribution of all variables according to each volunteers. (XLSX)
