# Correction: Assessment of Oral Conditions and Quality of Life in Morbid Obese and Normal Weight Individuals: A Cross-Sectional Study

**DOI:** 10.1371/journal.pone.0137707

**Published:** 2015-09-02

**Authors:** Joselene Martinelli Yamashita, Patrícia Garcia de Moura-Grec, Adriana Rodrigues de Freitas, Arsênio Sales-Peres, Francisco Carlos Groppo, Reginaldo Ceneviva, Sílvia Helena de Carvalho Sales-Peres

Affiliations 2 and 3 are incorrectly switched. The affiliations from Dr. Carlos Groppo and Dr. Reginald Ceneviva should appear as: Francisco Carlos Groppo ^2^ Reginaldo Ceneviva ^3^

**2** Department of Physiological Sciences, Piracicaba Dental School, University of Campinas, Piracicaba, São Paulo, Brazil **3** Department of Surgery and Anatomy, School of Medicine of Ribeirão Preto, University of São Paulo, Ribeirão Preto, SP, Brazil.
